# Parent Use and Efficacy of a Self-Administered, Tablet-Based Parent Training Intervention: A Randomized Controlled Trial

**DOI:** 10.2196/mhealth.5202

**Published:** 2016-04-20

**Authors:** Susan M Breitenstein, Louis Fogg, Edith V Ocampo, Diana I Acosta, Deborah Gross

**Affiliations:** ^1^ Rush University College of Nursing Chicago, IL United States; ^2^ Johns Hopkins University School of Nursing Baltimore, MD United States

**Keywords:** Internet, intervention, mobile app, mobile health, parenting, prevention

## Abstract

**Background:**

Parent training programs are traditionally delivered in face-to-face formats and require trained facilitators and weekly parent attendance. Implementing face-to-face sessions is challenging in busy primary care settings and many barriers exist for parents to attend these sessions. Tablet-based delivery of parent training offers an alternative to face-to-face delivery to make parent training programs easier to deliver in primary care settings and more convenient and accessible to parents. We adapted the group-based Chicago Parent Program (CPP) to be delivered as a self-administered, tablet-based program called the *ez* Parentprogram.

**Objective:**

The purpose of this study was to (1) assess the feasibility of the *ez* Parentprogram by examining parent satisfaction with the program and the percent of modules completed, (2) test the efficacy of the *ez* Parentprogram by examining the effects compared with a control condition for improving parenting and child behavior in a sample of low-income ethnic minority parents of young children recruited from a primary care setting, and (3) compare program completion and efficacy with prior studies of the group-based CPP.

**Methods:**

The study used a two-group randomized controlled trial (RCT) design with repeated measures follow up. Subjects (n=79) were randomly assigned to an intervention or attention control condition. Data collection was at baseline and 12 and 24 weeks post baseline. Parents were recruited from a large, urban, primary care pediatric clinic. *ez* Parentmodule completion was calculated as the percentage of the six modules completed by the intervention group parents. Attendance in the group-based CPP was calculated as the percentage of attendance at sessions 1 through 10. Satisfaction data were summarized using item frequencies. Parent and child data were analyzed using a repeated measures analysis of variance (RM-ANOVA) with simple contrasts to determine if there were significant intervention effects on the outcome measures. Effect sizes for between group comparisons were calculated for all outcome variables and compared with CPP group based archival data.

**Results:**

*ez* Parentmodule completion rate was 85.4% (34.2/40; 95% confidence interval [CI] = 78.4%-93.7%) and was significantly greater ( *P*<.05) than face-to-face CPP group attendance (135.2/267, 50.6%) attendance of sessions; 95% CI = 46.8%-55.6%). *ez* Parentparticipants reported the program as very helpful (35/40, 88.0%) and they would highly recommend the program (33/40, 82.1%) to another parent. *ez* Parentparticipants showed greater improvements in parenting warmth (F1,77 = 4.82, *P*<.05) from time 1 to 3. No other significant differences were found. Cohen’s d effect sizes for intervention group improvements in parenting warmth, use of corporal punishment, follow through, parenting stress, and intensity of child behavior problems were comparable or greater than those of the group-based CPP.

**Conclusions:**

Data from this study indicate the feasibility and acceptability of the *ez* Parentprogram in a low-income, ethnic minority population of parents and comparable effect sizes with face-to-face delivery for parents.

## Introduction

### Background

Behavioral, social, and emotional difficulties that begin early in life have long-term learning, academic, and relational consequences [ 1- 3]. Chronic pediatric mental health problems are among the top five disabilities affecting children in the United States and now more prevalent than childhood physical disabilities [ 4, 5]. Importantly, many of these social, emotional, and behavioral problems begin in the preschool years [ 2].

Prevention and intervention in the preschool years is critical before the behavior problems become fixed and disabling [ 6, 7]. Pediatric primary care clinicians are often the first professionals that families solicit regarding parenting concerns or behavioral problems in their children [ 8, 9]. Although a significant need exists in pediatric primary care for programs that teach effective parenting and prevent behavior problems these programs are not readily accessible to clinicians and parents. The purpose of this study was to examine the use and efficacy of a tablet-based parent training program (the *ez* Parentprogram) in a sample of parents recruited from a pediatric primary care setting.

Parent training (PT) - a set of systematic programs for teaching parents child management skills - is widely used to promote positive parenting and reduce behavioral risk in young children [ 10, 11]. Early prevention efforts focused on PT are important because parent behavior is a modifiable risk factor for early child behavior problems and improving positive and skilled parenting is a powerful predictor of positive child outcomes [ 10, 12, 13]. Though PT programs have been shown to be effective, there are two important limitations to these programs commonly delivered in face-to-face group or individual settings. These limitations include low parent participation rates, particularly among low-income parents, and implementation challenges in existing primary health care systems.

Parent attendance rates for those who enroll in PT typically range 35% to 50% of sessions and up to one-third who sign up attend no sessions [ 14, 15]. Primary logistic barriers to enrollment and attendance in group-based PT include lack of time, childcare, schedule conflicts, and competing demands [ 14, 16]. For low-income families, these barriers may be magnified by concerns with transportation, childcare, and neighborhood safety; as well as demands from work, family and friends, high stress, and poor health.

In addition to parent participant barriers, there are practice barriers. Although pediatric primary care is an ideal venue for programs for improving parenting and preventing behavior problems, there are limited treatment and prevention options that fit into the primary care setting [ 17]. Indeed, PT programs are traditionally delivered in face-to-face formats (either individually or in group-based settings) and require trained facilitators and weekly parent attendance. Implementing multiple face-to-face sessions of PT is challenging in a busy primary care setting. For instance, a typical well-child visit lasts 8 to 18 minutes during which time clinicians are expected to complete activities including a physical exam, assessment of developmental milestones met, assessment of the child’s nutritional status, and deliver any treatments and immunizations. Providing in depth parenting guidance to families struggling with children’s emotional or behavioral problems may be unrealistic [ 18, 19]. Further, clinicians’ time for pediatric visits related to behavioral problems is reimbursed at a significantly lower rate than general medical visits [ 19]. The use of Internet-based methods to deliver PT is a feasible and potentially cost-effective approach to address the challenges of delivering PT in primary care settings. Internet delivery addresses issues related to parent participation by increasing accessibility, availability, and parent-controlled access.

### The *ez* PARENTProgram

**Figure 1:**
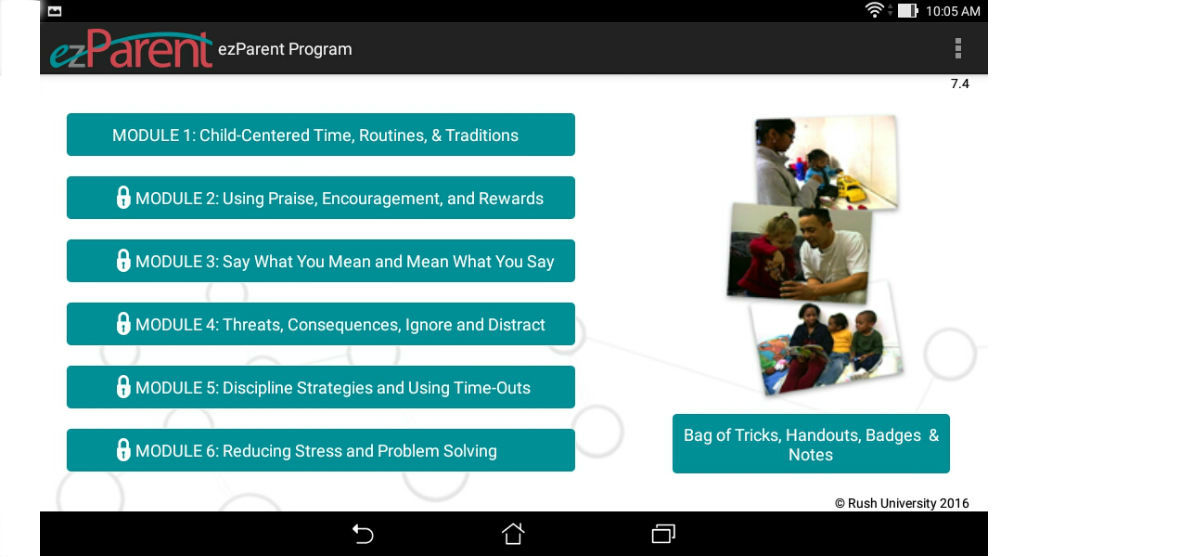
ezPARENT program home page and module topics.

The *ez* Parentprogram is a delivery adaptation of the evidence-based, group delivered Chicago Parent Program (CPP). The program is a prevention intervention designed to promote parenting competence and prevent child behavior problems in children 2- to 5-years old [ 20, 21]. Like the group-based program, the six modules of the *ez* Parentprogram teach parents evidence-based strategies for encouraging good behavior and decreasing misbehavior in children. See Figure 1for a list of module content. The *ez* ParentProgram was originally named the *electronic*CPP and changed to the *ez* ParentProgram in 2016. For a full description of the development of the program see Breitenstein et al [ 20].

The *ez* Parentprogram is a self-administered program that is downloaded onto an Android tablet computer as an app. It is one of the first adaptations of an evidence-based group delivered program to incorporate tablet-based technology. The program is set up so that users move sequentially through the modules (ie, parents must complete the first module prior to the second module being unlocked). Once a module is unlocked, parents can access the module and return to any portion of that module at a later time. We estimate that initial completion of one module would take approximately 1 hour [ 20].

Like other PT programs, rather than focusing primarily on education or increasing knowledge, the *ez* Parentprogram assists parents in acquiring a range of evidence-based parenting skills [ 13, 20]. There are several unique aspects of the *ez* Parentprogram developed to promote parent engagement and support learning. These are (1) video vignettes of parents interacting with their children, which support vicarious learning and present examples of the various parenting strategies, (2) knowledge questions, which assess parent understanding of the module content and provide added information when needed, (3) interactive “game” activities, which are intended to be fun and keep parents stimulated and engaged, and (4) module practice assignments, which provide opportunities for parents to practice what they are learning with their children [ 20].

Consistent with the group-based CPP, the *ez* Parentprogram is designed to be culturally and contextually relevant for low-income, ethnically diverse parents of young children [ 21]. During the development of the tablet program, we worked with low income and ethnic minority parents to assure the content was relevant and that the app was easy to use, attractive, and interesting [ 20]. The use of mobile technology allowed us to design the program to be interactive and accessible, a particularly salient feature for African American and Hispanic parents who are among the most active users of the mobile Internet [ 22, 23]. Therefore, parent training via an app on mobile devices may be an ideal method to increase the reach and accessibility of parent training programs, particularly among low-income African-American and Hispanic families.

### Study Purpose and Hypothesis

The purpose of this study was to establish the feasibility and efficacy of the *ez* Parentprogram. To examine feasibility, we examined parent satisfaction with the program and the percent of modules completed relative to percent of face to face sessions attended in prior studies of the group-based CPP. To test efficacy, we examined the effect of the *ez* Parentprogram against an attention control condition for improving parenting and child behavior in a sample of low-income ethnic minority parents of young children recruited from a primary care setting. We hypothesized that the percent of *ez* Parentmodules completed would be comparable to or greater than the percent of group-based CPP session completed; compared with an attention control group, parents in the *ez* Parentintervention group would report using more positive discipline strategies, greater parenting self-efficacy, and decreased parenting stress and child behavior problems; and effect sizes for improvement in parent and child behaviors would be comparable to or greater than the group-based CPP.

## Methods

### Trial Design

The study used a two-group randomized control trial (RCT) design with repeated measures follow-up. Subjects were randomly assigned to either the intervention ( *ez* Parentgroup) or attention control condition (health promotion group). Data collection in both conditions was at baseline and at 12 and 24 weeks post baseline. This study was reviewed and approved by the University’s institutional review board.

Parents in the *ez* Parentgroup received the six module *ez* Parentprogram to be completed over a 12-week period (allowing approximately 2 weeks per module). During the same time period, parents in the health promotion control group received information on various topics (ie, nutrition, exercise, finance, safety, medical information, and entertainment) via a website that included links and portable document format handouts for each topic. These materials were the same health promotion handouts and websites distributed at the primary care pediatric recruitment site. The health promotion condition was designed to control for attention related to technology use over the same period of time.

### Sample

Parents were eligible to participate in the study if they were the parent or legal guardian of a child between the ages of 2 to 5 years, the child was receiving or eligible to receive Medicaid insurance (eg, All Kids Assist or Share in Illinois), and the child received medical care at the pediatric primary care recruiting site. Only one parent per family participated in the study. If a parent had more than one child between the ages of 2 and 5 years, the parent selected one child to report on their behavior over the length of the study. Currently, the *ez* Parentprogram is only available in English; therefore, parents who were unable to speak and read English were excluded.

Parents were recruited between October 2013 and June 2014 from a large, urban, primary care pediatric clinic located on the near west side of Chicago. According to the clinic, most of the families they serve are African American (50%) or Latino (30%) and over 65% of families have low incomes or receive Medicaid. We advertised broadly at the primary care site using project flyers that contained information about the purpose of the study, inclusion criteria, expectations for participation, and contact information for parents to directly contact the study staff. An interest form was included in the flyer; parents completed the form indicating their willingness to be contacted by the study staff. If a parent was eligible and interested in participating, then a 2-hour meeting was scheduled in our research offices to complete the participant consent, baseline data collection, and randomization to condition.

**Figure 2:**
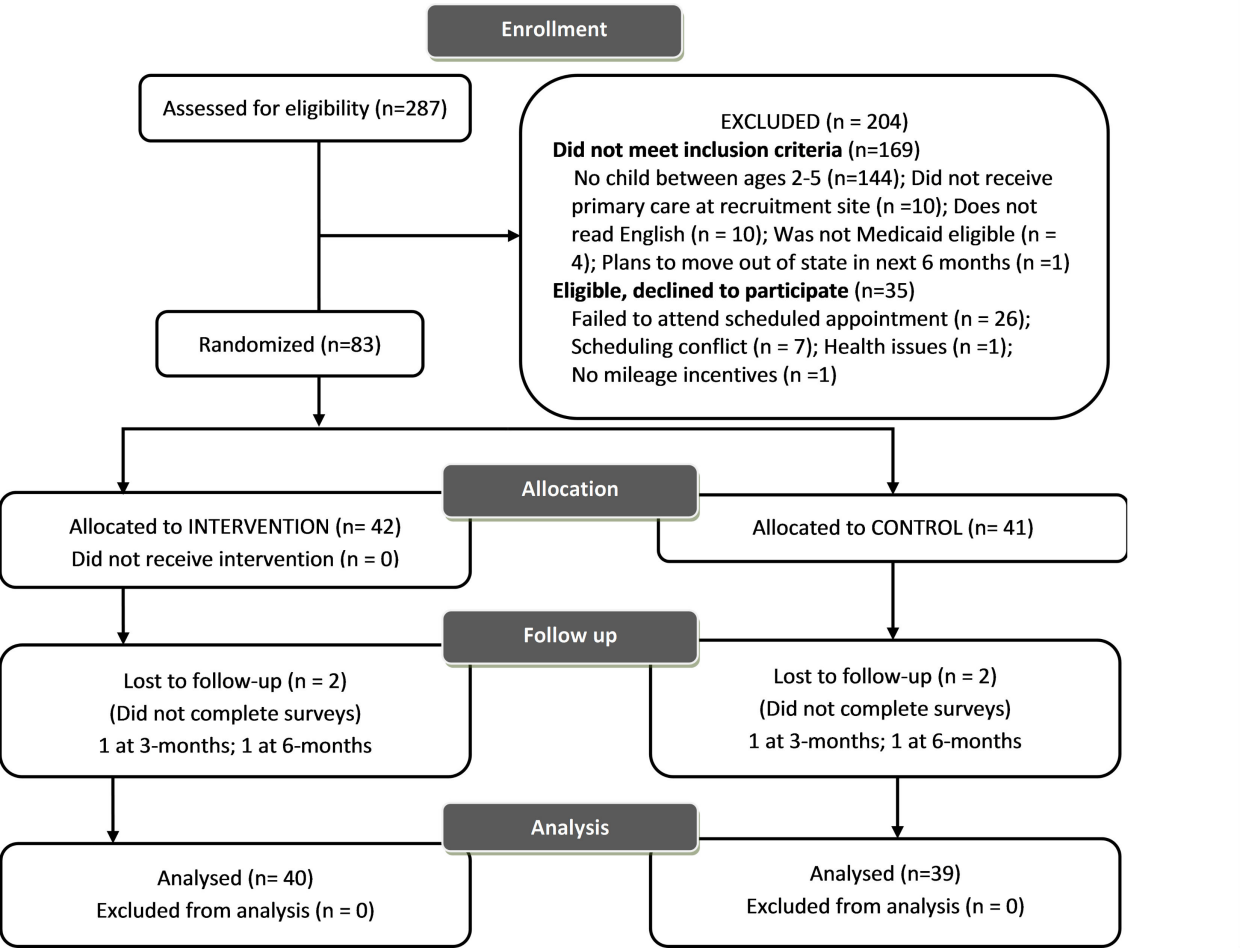
CONSORT flow diagram.

Figure 2depicts the participant flow in the trial. Two hundred eighty-seven parents were assessed for eligibility. Of the 118 parents eligible for the study, 70.3% (83/118) of parents consented to participate, completed baseline assessments, and were randomized to either the *ez* Parentor health promotion control condition. Of the 83 parents randomized, 79 parents completed follow-up assessments (95.2% retention rate).

The sample was predominantly comprised of single, ethnic minority (African-American or Hispanic) mothers of young children earning annual incomes of less than $20,000 (see Table 1). There were no significant demographic differences between the intervention and control groups.

### Variables and Measures

Module Completion and Satisfaction

Parents earn a “module badge” at the end of each module and after they review the practice assignment. After completion of the module, parents are alerted with a pop-up in the program congratulating them on earning the module badge. Parents can view their earned badges in the “my badges” section of the program. The *ez* Parentdigital platform provides time stamps of when parents earn the module badge. Intervention dose was defined by parent receipt of the module badge. For this analysis, we included badges that were earned prior to the 12 week post baseline data collection appointment corresponding with the intervention completion period (eg, 6 modules × 2 weeks per module).

Satisfaction was measured using an end of program survey administered 6 months after baseline. Parents rated how helpful they found the *ez* Parentprogram (very helpful, a little helpful, or not at all helpful), their overall satisfaction with the program (very dissatisfied, dissatisfied, satisfied, or very satisfied), and whether they would recommend the *ez* Parentprogram to other parents (not recommend, recommend, or highly recommend). Parents in the control group rated their perception of helpfulness (very helpful, a little helpful, or not at all helpful) of the information provided in the health promotion website (eg, nutrition, exercise, finances, safety, medical, and entertainment) and whether they would recommend the website to other parents (not recommend, recommend, or highly recommend).

**Table 1:** Study participant demographics (N=79; frequency [%]).

Demographic Variable		Full Sample N=79	Control n=39	Intervention n=40
		n (%)	n (%)	n (%)
**Child Age**				
	2-years old	18 (22.8%)	10 (25.6%)	8 (20.0%)
	3-years old	17 (21.5%)	10 (25.6%)	7 (17.5%)
	4-years old	29 (36.7%)	14 (35.9%)	15 (37.5%)
	5-years old	15 (19.0%)	5 (12.8%)	10 (25.0%)
**Child gender**				
	Female	45 (57.0%)	22 (56.4%)	23 (57.5%)
	Male	34 (43.0%)	17 (43.6%)	17 (42.5%)
**Relationship to child**				
	Mother	75 (94.9%)	37 (94.9%)	38 (95.0%)
	Foster Mother	1 (1.3%)	1 (2.6%)	--
	Grandmother	3 (3.8%)	1 (2.6%)	2 (5.0%)
**Parent age**				
	age 18-29	27 (34.2%)	15 (38.5%)	12 (30.0%)
	age 30-49	50 (63.3%)	23 (59.0%)	27 (67.5%)
	age 50+	2 (2.5%)	1 (2.6%)	1 (2.5%)
**Parent race/ethnicity**				
	African American	51 (64.6%)	28 (71.8%)	23 (57.5%)
	Hispanic	24 (30.4%)	10 (25.6%)	14 (35.0%)
	White/other ^a^	4 (5.1%)	1 (2.6%)	3 (7.5%)
**Parent education**				
	Less than high school	7 (8.9%)	2 (5.1%)	5 (12.5%)
	High school/GED	10 (12.7%)	6 (15.4%)	4 (10.0%)
	Some college/AD	49 (62.0%)	23 (59.0%)	26 (65.0%)
	College/Graduate school	13 (16.5%)	8 (20.5%)	5 (12.5%)
**Parent employment status**				
	Working	36 (46.2%)	21 (55.3%)	15 (37.5%)
	Not Working	42 (53.8%)	17 (44.7%)	25 (62.5%)
**Annual income**				
	< $20,000/yr	52 (65.8%)	24 (61.5%)	28 (70.0%)
	$20,000-$40,000/yr	22 (27.8%)	11 (28.2%)	11 (27.5%)
	> $40,000/yr	5 (6.3%)	4 (10.3%)	1 (2.5%)
**Marital status**				
	Married or domestic partnership	22 (27.8%)	12 (30.8%)	10 (25.0%)
	Never married	48 (60.8%)	21 (53.8%)	27 (67.5%)
	Divorced or separated	9 (11.4%)	6 (15.4%)	3 (7.5%)

^a^One parent identified as Cherokee Indian, German, Irish, and Italian.

#### Parent Outcomes

Parenting self-efficacy, behavior, and stress were assessed using self-report measures. The 38-item Toddler Care Questionnaire (TCQ) was used to measure parenting self-efficacy [ 24]. The TCQ measures parent self-efficacy in managing situations and tasks that are specific to raising young children. TCQ scale scores range from 38 (not at all confident) to 190 (very confident). Reliability (Cronbach’s alpha) for the TCQ for this sample was 0.94.

Parent discipline strategies were measured using the 40-item Parenting Questionnaire (PQ) [ 25, 26]. The PQ includes three discipline scales measuring parental warmth (Warmth), extent to which they follow through on discipline (Follow Through), and use of corporal punishment (Corporal Punishment). Parents rate each item on a scale of 1 (almost never) to 5 (very often). Reliability (Cronbach’s alpha) for the PQ scales were 0.88 (Warmth), 0.81 (Follow Through), and 0.66 (Corporal Punishment).

The 36-item Parenting Stress Index-Short Form (PSI-SF) was used to measure parenting stress. The PSI-SF is derived from the validated 101-item PSI and mirrors the strong validity of the PSI [ 27]. Parents respond to items on a 5-point Likert scale ranging from 1 (strongly disagree) to 5 (strongly agree) resulting in a total stress score. Higher scores on the PSI-SF indicate higher parenting stress. The PSI-SF also includes a published cut-off score for clinically significant stress based on scores above the 85th percentile [ 28]. Reliability (Cronbach’s alpha) for the PSI-SF for this sample was 0.92.

#### Child Behavior Problems

Parents reported child behavior problems using the 36-item Eyberg Child Behavior Inventory (ECBI) [ 29]. The ECBI is for parents of children ages 2- to 16-years old and assesses problem behavior on two scales, the Intensity Scale and the Problem Scale. The Intensity Scale assesses the frequency of 36 problem behaviors on a 7-point scale ranging from 1 (the behavior never happens) to 7 (the behavior is always happening). The Problem Scale assesses parent perception of each of the behaviors as being problematic for him or her as a parent (yes or no). The ECBI includes cut off scores for each scale indicative of clinical significant child behavior problems based on scores that are 1.5 standard deviations (SD) above the mean (the 93rd percentile) [ 29]. Reliability (Cronbach’s alpha) for the ECBI for this sample was 0.92 (Intensity Scale) and 0.93 (Problem Scale).

#### Group-Based CPP Archival Data

The data obtained in this study were compared with group-based CPP archival data collected on a racially, ethnically, and socioeconomically comparable sample of 267 parents that received the intervention as part of a RCT [ 15]. There were no significant differences between the group-based intervention group (n=267) and the *ez* Parentintervention group (n=40) for income or race. Data were compared on intervention dose (ie, percent of *ez* Parentmodule completion versus percent of face-to-face parent sessions attended) and effect size changes in parenting self-efficacy (TCQ), parent discipline strategies (PQ), and child behavior (ECBI). Comparison of the *ez* Parentand group-based CPP effect size estimates is important in establishing the comparability of the magnitude of treatment effects of the intervention using different delivery methods. The group-based CPP includes 12, 2-hour group sessions facilitated by two trained leaders [ 21]. The six modules of the *ez* Parentprogram parallel the content of the first 10 sessions of the group-based CPP (see Textboxes 1and 2) [ 20]. The 11th and 12th group sessions do not include new content and are used to help parents synthesize the knowledge and skills acquired from the prior sessions, these sessions correspond with the review materials included in the *ez* ParentProgram.

**ezParent program.**

Module 1: child-centered time, routines and traditions

Module 2: using praise, encouragement, and rewards

Module 3 **:**say what you mean and mean what you say

Module 4: threats, consequences, ignore and distract

Module 5: discipline strategies and using time-outs

Module 6: reducing stress and problem solving

Module Reviews: summary materials, handouts, and ability to return to any module content

**CPP-group based topics.**

Week 1: child-centered time

Week 2: family routines and traditions

Week 3: using praise and encouragement

Week 4: using rewards for challenging behaviors

Week 5: say what you mean and mean what you say

Week 6: threats and consequences

Week 7: using ignore and distract

Week 8: using time-outs

Week 9: reducing your stress

Week 10: problem solving

Week 11: putting it all together (session 1-10 review)

Week 12: booster session (1-2 months later)

### Procedures

At their first study appointment, parents were enrolled in the study by completing the informed consent process and responding to all demographic and survey instruments. Consents and survey data were collected on an Internet-based data collection app developed for this study. The goal of using Internet-based data collection was to increase the efficiency and ease of data collection and entry. Prior to recruitment, study staff tested the data collection app for ease of use and accuracy of data collection. Once established, the data collection app was launched. Total time for completion of the surveys at baseline was 35 to 40 minutes. Randomization to group assignment was made using stratified random sampling based on time sequential cohorts. The randomization table was built into the data collection app and group assignment was made after all baseline data were acquired. Parents were assured that the study assignment was random and had no connection with their responses to the survey questions. After study assignment was revealed, all parents in the control and intervention groups were given Android tablets to use for the duration of the study.

### Intervention Fidelity

Several strategies were implemented to ensure intervention fidelity (eg, strategies to monitor and enhance the reliability and validity of the intervention) [ 30]. After study assignment was revealed, the study staff provided a structured training (using written and verbal information) to all participants on how to use the tablet. Additional training was then provided specific to assigned condition. Following training, parents were prompted to verbalize their understanding and conducted return demonstrations to assess ability to use the tablet and the health promotion website (control) or *ez* Parentapp (intervention).

Parents in the *ez* Parentcondition received automated text message reminders to complete the *ez* Parentprogram if they had not completed the module content. Automated text messages also included positive encouragement regarding completion of the program and of practice assignments. These messages were consistent across all intervention parents.

### Analytic Approach

Parent participants were included in the analysis nondependent on their adherence or nonadherence to the *ez* Parentprogram. Parents (n=4) who were lost to follow up after enrollment were not included because we were unable to collect any follow up data for outcome analysis (see Figure 2). Parent completion of the *ez* Parentmodules was calculated as the percentage of the six modules completed by each of the intervention group parents (n=40). For comparison to the group-based CPP, intervention attendance was calculated as the percentage of sessions 1 through 10 of the CPP group attended by intervention group parents (n=267). This analysis is based on content completion not on the amount of time spent in either the group-based or tablet-based program. Content across the *ez* Parentmodules and the CPP group sessions is comparable (see Textboxes 1and 2). The *ez* Parentand group based rates of participation were compared using 95% confidence intervals (CI). Satisfaction data were summarized using item frequencies.

Chi square tests were used to compare baseline data between parents in the *ez* Parentand health promotion control group and determine if there were significant differences. Parent and child data were analyzed using a repeated measures analysis of variance (RM-ANOVA) with simple contrasts to determine if there were significant intervention effects on the outcome measures from baseline to 12 weeks post baseline (T1 to T2) and from baseline to 24 weeks post intervention (T1 to T3).

Effect sizes for between group comparisons were calculated for all outcome variables using Cohen’s d [ 31]. For comparison to the group-based CPP, we calculated effect sizes from data published by Breitenstein and colleagues [ 15] (effect size comparisons for the PSI-SF are not included as this measure was not part of the group-based evaluation). Effect sizes were calculated T1 to T2 and for T1 to T3 using the following equation:

(xİ _*t1*_−xİ _*t2*_) − (xİ _*c2*_−xİ _*c1*_) /SD _*pooled*_

wherexİ _*t2*_= treatment group mean at time 2;xİ _*t1*_= treatment group mean at time 1;xİ _*c2*_= control group mean at time 2;xİ _*c1*_= control group mean at time 1; and SD _*pooled*_= the pooled SDs of all reported groups (eg, treatment and control) [ 31].

## Results

### *ez* PARENTModule Completion

Table 2presents the percent of the sample completing each module (ie, earned a module badge) during the first 3 months of the study and the percent of parents in the archival comparison sample attending the face to face CPP group-based sessions. All parents in the *ez* Parentsample earned the module 1 completion badge. Across all six modules, the average *ez* Parentmodule completion by parent was 85.4% (34/40) of modules (95% CI = 74.5%-96.4%). In contrast, average parent attendance for the face-to-face CPP groups was 50.6% (135/267) of sessions (95% CI = 44.6%-56.6%), and 24% (64/267) of the archival CPP sample failed to attend any sessions [ 15]. The *ez* Parentdose rate was higher than the upper CI for the group CPP dose, suggesting that parent participation rates of the *ez* Parentand group based CPP were significantly different ( *P*<.05).

**Table 2:** *ez* Parentmodule (n=40) and CPP group-base (n=267) Dose.

*ez* PARENTmodule	Corresponding CPP group session(s)	*ez* PARENTmodule completion n (%)	CPP group attendance n (%)
Module 1	Sessions 1-2	40 (100%)	157 (58.8%)
Module 2	Sessions 3-4	39 (97.5%)	143 (53.6%)
Module 3	Session 5	38 (95.0%)	134 (50.2%)
Module 4	Sessions 6-7	33 (82.5%)	133 (49.6%)
Module 5	Session 8	29 (72.5%)	120 (44.9%)
Module 6	Sessions 9-10	26 (65.0%)	124 (46.4%)

### Satisfaction

Of the parents in the *ez* Parentgroup, 87.5% (35/40) reported the *ez* Parentprogram being very helpful and 12.5% (5/40) a little helpful. In the control group parent reports of helpfulness of the topics on the health promotion website (control group) ranged from 46.2% (18/39) to 74.4% (29/39) very helpful; 20.5% (8/39) to 28.2% (11/39) a little helpful; and 2.6% (1/39) to 5.1% (2/39) not at all helpful. The nutrition topic in the website was the highest ranked as very helpful. Of parents in the *ez* Parentcondition, 42.5% (17/40) reported that it was a little bit hard to regularly use the *ez* Parentprogram, 2.5% (1/40) very hard, and 52.5% (21/40) not at all hard. Satisfaction with the *ez* Parentprogram was high, with 80.0% (32/40) of parents reporting they were very satisfied and 20.0% (8/40) reporting they were satisfied with the program.

Parents in the *ez* Parentgroup were more likely to recommend the *ez* ParentProgram to other parents than parents recommending the health promotion site (Kendall’s *Tau-b*= 0.25; *P*<0.05). Overall, 82.5% (33/40) of the *ez* Parentgroup reported they would highly recommend, 15.0% (6/40) would recommend, and 2.5% (1/40) didn’t know if they would recommend the program to another parent. In contrast, 59.0% (23/39) of parents in the health promotion control group reported they would highly recommend *,*35.9% (14/39) would recommend, 2.6% (1/39) would not recommend, and 2.6% (1/39) didn’t know if they would recommend the website to another parent.

### Parent and Child Outcomes

Table 3presents means and SDs for each variable by condition at each data collection time point. At baseline, there were no significant differences between the *ez* Parentand health promotion control groups for parent behavior (PQ), self-efficacy (TCQ), and stress (PSI-SF), or child behavior (ECBI). At baseline, 6.3% (5/79) of the sample scored above the PSI cut off score (>109) [ 28]; 25.3% (20/79) scored above the ECBI problem scale cut off score (>14); and 19% (15/79) of the sample scored above the ECBI intensity scale cut-off score (>130) [ 29].

From T1 to T3, there was a significant difference between conditions for parenting warmth on the PQ ( *F*_1,77_= 4.82, *P*<.05). There were no significant differences between the intervention and control conditions on parents’ reports of their follow through on discipline, use of corporal punishment, parenting stress, parenting self-efficacy, or child behavior problems.

In Table 4, we present effect size estimates for the *ez* Parentprogram and comparison effect sizes for the group-based CPP [ 15]. A Cohen’s d of .2 is considered a small effect, .5 a medium effect, and .8 or larger a large effect [ 31]. Effect sizes from T1 to T3 were in the small range for parent warmth (PQ), parent corporal punishment (PQ), follow through (PQ), parenting stress (PSI-SF), and child behavior problems based on the ECBI intensity scale. With the exception of parenting self-efficacy (TCQ) and corporal punishment (PQ), *ez* Parenteffect sizes were comparable or greater than those of the group-based CPP.

**Table 3:** Mean and standard deviation of outcome variables for intervention (n=40) and control (n=39) groups.

		Assessment Time Point		
Variable		Time 1 (T1) ^a^ M (SD)	Time 2 (T2) ^b^ M (SD)	Time 3 (T3) ^c^ M (SD)
**Parent warmth (PQ ^d^)**				
	Intervention	94.23 (7.96)	95.15 (7.68)	95.73 (7.34)
	Control	95.08 (10.04)	95.39 (7.43)	93.67 (11.71)
**Parent corporal punishment (PQ)**				
	Intervention	6.00 (2.00)	5.85 (2.4)	5.58 (2.16)
	Control	6.36 (2.58)	5.92 (2.22)	6.26 (2.45)
**Parent follow through (PQ)**				
	Intervention	20.18 (5.32)	21.48 (4.65)	21.65 (5.13)
	Control	18.85 (4.94)	19.00 (5.27)	19.36 (5.67)
**Parenting self-efficacy (TCQ ^e^)**				
	Intervention	165.50 (17.12)	167.70 (14.43)	169.13 (14.81)
	Control	163.44 (21.69)	167.31 (23.37)	164.51 (23.94)
**Parenting stress (PSI-SF ^f^)**				
	Intervention	75.03 (20.30)	70.24 (19.32)	67.90 (17.34)
	Control	75.89 (19.00)	70.95 (20.98)	72.46 (20.61)
**Child behavior problems (ECBI ^g^)**				
	Intervention	7.41 (6.59)	5.78 (6.06)	5.50 (5.74)
	Control	10.08 (8.79)	7.18 (7.59)	8.11 (8.85)
**Child behavior intensity (ECBI)**				
	Intervention	103.55 (28.94)	96.68 (29.96)	94.88 (26.89)
	Control	104.79 (29.59)	98.04 (27.92)	101.23 (30.74)

^a^Baseline.

^b^3 months post baseline.

^c^6 months post baseline.

^d^Abb: Parent Questionnaire.

^e^Abb: Toddler Care Questionnaire.

^f^Abb: Parenting Stress Index-Short Form.

^g^Abb: Eyberg Child Behavior Inventory.

**Table 4:** Comparison of between group effect size estimates of *ez* ParentProgram (n=40 control; n=39 intervention) and group CPP (n=237 control; n=267 intervention).

	Effect Sizes			
Variable	Time 1-2 ^a,b^		Time 1-3 ^a-c^	
	*ez* Parent	Group CPP ^a^	*ez* Parent	Group CPP ^d^
Parent warmth (PQ ^e^)	0.07	-0.06	0.31	0.10
Parent corporal punishment (PQ ^e^)	-0.13	0.15	0.14	0.26
Parent follow through (PQ)	0.23	0.17	0.18	0.08
Parenting self-efficacy (TCQ ^f^)	-0.09	0.21	0.13	0.22
Parenting stress (PSI-SF ^g^)	-0.01	-- ^b^	0.19	-- ^i^
Child behavior problems (ECBI ^h^)	-0.18	-0.06	-0.01	0.05
Child behavior intensity (ECBI)	0.00	0.19	0.18	0.20

^a^Baseline.

^b^12 weeks post baseline.

^c^24 weeks post baseline.

^d^Effect sizes estimated from data in Breitenstein et al [ 15].

^e^Abb: Parent Questionnaire.

^f^Abb: Toddler Care Questionnaire.

^g^Abb: Parenting Stress Index;

^h^Abb: Eyberg Child Behavior Inventory.

^i^PSI-SF was not reported in Breitenstein et al [ 15].

## Discussion

### Principal Findings

In this study we evaluated the feasibility, acceptability, and preliminary efficacy of the tablet-based adaptation of the CPP, the *ez* Parentprogram, in a sample of low-income, ethnic minority parents of 2- to 5-year-old children seen in primary care. The CPP is an evidence-based parenting skills program originally designed to be delivered in a face-to-face parent group format [ 21]. However, like many preventive interventions targeting low-income families that require face-to-face involvement, participation rates have typically been low [ 14, 16]. Moreover, pediatric primary care providers, who serve a large population of families receiving Medicaid, are often unable to devote the time needed to provide parenting skills training and support. These problems diminish the impact and reach of evidence-based parenting programs. Given the challenges of using face-to-face formats for parenting skills training, we were particularly interested in examining the amount of intervention (ie, dose) parents would receive using a self-administered digital format for acquiring parenting skills training compared with a face-to-face group format.

Overall, the results indicate that the *ez* Parentprogram is feasible and acceptable in a low-income, ethnic minority population of parents. Compared with a control group of parents using a health promotion website, *ez* Parentprogram parents were more likely to report that they would recommend the program to another parent. Compared with a comparable archival sample of parents enrolled in the face to face group-based CPP, parents enrolled in *ez* Parentreceived a higher dose of parenting content. Specifically, parents in the *ez* Parentgroup completed over 85.0% (34/40) of the modules compared with parents in the face-to-face format who attended on average only 50.6% (135/267) of the parent group sessions. Moreover, all of the *ez* Parentparticipants completed at least one module whereas 24.0% (64/267) of parents enrolled in the face-to-face group format, never attended a single session. These findings are consistent with a recent review of digitally delivered parenting interventions reporting that 41.7% to 99.2% of participants completed their digitally delivered parent training interventions [ 32]. Furthermore, these data demonstrate that a digital format for delivering parenting skills training to a predominantly low-income ethnic minority population of parents is feasible and acceptable, and may greatly extend the reach for helping more parents and young children in primary care.

Although module completion rates in this study were promising, there is more work to be done to understand patterns of parent use of the *ez* Parentprogram. A benefit of using technology is the ability to track all aspects of parent usage of the app. Digital tracking data can provide important information regarding parent behavior in interacting with the intervention, specifically, how and when parents use the *ez* Parentprogram and what content they access. For example, information regarding the number of visits parents make to components of the program, time stamps of visits to each portion of the program, and the frequency which parents return to completed content. In future work, we will conduct detailed analysis of this usage data. Indeed, research of Internet-delivered interventions is beginning to emerge related to patterns of program use and intervention outcome [ 33, 34]. A fine tune analysis of usage metrics will expand our knowledge related to dose and program effectiveness.

Overall, we found the effects of the *ez* Parentprogram on parenting and child behavior problems were in the small range and many were comparable to the effect sizes obtained from archival samples using the CPP in the face-to-face format. Although significant improvements were found for parent use of warmth in their discipline, no improvements were found for parent use of corporal punishment, following through on discipline, parenting stress, and child behavior problems relative to the control group. There are three possible reasons for these results. First, the power calculation for this study was based on estimated effect sizes for intervention dose rather than parent and child outcomes. Much larger sample sizes would be needed to detect significant differences based on small effects, which are typical for prevention studies [ 35, 36]. Therefore, we may not have adequate power to detect differences between the intervention and control parents in this analysis.

Second, although we recruited from a population of families with social risk factors related to parent and child dysfunction (eg, low income), the parents in our study reported low levels of parenting stress and relatively few child behavior problems at baseline. Less than one-quarter of the parents reported child behavior problems exceeding the cut point for clinically significant problems and less than 6.3% (5/79) reported clinically significant levels of parenting stress. Therefore, floor effects in this relatively healthy population may have made it difficult to detect improvements over time. Greater effects may be more detectable in a referred population of parents and young children.

Third, the lack of effects may be due to the limited amount of time allotted for assessing parent and child behavior change. It is possible that more time for parents to absorb the new information, practice the new skills, and observe changes in themselves and their children may yield greater improvements in discipline strategies, parenting self-efficacy, and child behavior.

### Study Limitations and Future Directions

Although our findings indicate feasibility and acceptability of the *ez* Parentprogram and comparable effect sizes to face-to-face delivery for parents recruited from primary care we recruited from one location from a relatively healthy population. It is possible that parents involved in this study were highly motivated to participate and learn from the intervention. Future studies of the *ez* Parentprogram will employ a larger sample size from multiple primary care sites and longer term follow-up to establish the efficacy of the *ez* Parentprogram in modifying parent and child behaviors. In addition, to lay the ground work for full scale implementation in primary care sites, implementation, and cost effectiveness of the *ez* Parentprogram in primary care will be determined.

Using an archival sample for a comparison group of delivery methods presents methodological limitations because of the lack of experimental control across the two groups. Although not a purpose of the original evaluation of the *ez* Parentprogram, we felt it was important to demonstrate an initial comparison of dose and outcome to the group-based CPP. Our analysis suggests they may be comparable but a true test would require a randomized design comparing group-based CPP to *ez* Parent. Indeed, a RCT structured as an equivalence or noninferiority trial with an adequately powered sample size would allow a head to head comparison of the two formats, directly compare intervention outcomes and dose, and allow us to understand which parents are likely to benefit from each delivery format.

Another potential limitation is the use of parent report to evaluate the intervention. A more robust measure of intervention efficacy could include multi-informant and multimethod data. However, previous findings from RCTs of the group-based CPP have found significant findings across three sources of data: parent self-report, teacher report, and parent-child observation [ 15, 37]. In these studies, the parent self-report findings have consistently agreed with other methods of measuring parent and child outcomes.

It is important to understand how parents use the information and program. In this analysis we assessed one metric of program usage – module completion. Although module completion provides an overall picture of parent dose it does not provide us with specific information related to patterns of use, time spent on the program, and other important data of *ez* Parentuse. In addition, we assessed program use from baseline to the 12-week follow-up even though parents had access to the program throughout the 24-week study period. Therefore, as previously noted, an analysis of usage data is critical in understanding patterns using the *ez* Parent intervention and parent’s ongoing use of information after the initial module completion. This information will help understand the change mechanisms of the intervention. Further, demonstrating the processes and patterns related to using tablet-based behavior change interventions is important to the field of eHealth behavioral interventions.

## Abbreviations

CI: confidence interval
CPP: Chicago Parent Program
ECBI: Eyberg child behavior inventory
PT: parent training
PQ: parenting questionnaire
RCT: randomized controlled trial
RM-ANOVA: repeated measures analysis of variance
PQ: parent questionnaire
PSI-SF: parenting stress index-short form
SD: standard deviation
T1: baseline
T2: 3 months post baseline T3: 6 months post baseline
TCQ: toddler care questionnaire
